# Supplementary material for: Observation of the modification of quantum statistics of plasmonic systems
Source: Nat Commun. 2021 Aug 27;12:5161. doi: 10.1038/s41467-021-25489-4 (PMC8397772; doi:10.1038/s41467-021-25489-4)
Supplement: Supplementary file 1 — Supplementary Information [file 41467_2021_25489_MOESM1_ESM.pdf]

Observation of the Modification of Quantum Statistics of Plasmonic Systems: Supplementary Information

Chenglong You,[1] Mingyuan Hong,[1] Narayan Bhusal,[1] Jinnan Chen,[2] Mario A. Quiroz-Juárez,[3] Joshua Fabre,[1] Fatemeh Mostafavi,[1] Junpeng Guo,[2] Israel De Leon,[4] Roberto de J. León-Montiel,[5] and Omar S. Magaña-Loaiza[1]

[1]*Quantum Photonics Laboratory, Department of Physics & Astronomy, Louisiana State University, Baton Rouge, LA 70803, USA*

[2]*Department of Electrical and Computer Engineering, University of Alabama in Huntsville, Huntsville, Alabama 35899, USA*

[3]*Departamento de Física, Universidad Autónoma Metropolitana Unidad Iztapalapa, San Rafael Atlixco 186, 09340 Ciudad México, México*

[4]*School of Engineering and Sciences, Tecnológico de Monterrey, Monterrey, Nuevo Leon 64849, Mexico*

[5]*Instituto de Ciencias Nucleares, Universidad Nacional Autónoma de México, Apartado Postal 70-543, 04510 Cd. Mx., México*

(Dated: August 12, 2021)

This document provides supplementary information to "Observation of the Modification of Quantum Statistics of Plasmonic Systems". We provide the details of our theoretical model, the statistical properties of our optical sources, additional data for our experiment, and the characterization of our sample.

SUPPLEMENTARY NOTE 1. THEORY DESCRIPTION

Let us start by noticing from Fig. 1(b) of the main manuscript that the detected field after the slits is the result of three contributing sub-fields. The first two contributions correspond to the horizontally- and vertically-polarized fields that traverse the illuminated slit, with mean photon numbers $\eta\bar{n}_{\mathrm{s}}$, and $(1-\eta)\bar{n}_{\mathrm{s}}$, respectively. Note that $\bar{n}_{\mathrm{s}}$ is the total mean photon number of the field after it has traversed the slit and $\eta = \cos^2\theta$, with θ describing the polarization angle of the initial illuminating photons, defined with respect to the vertical axis. The third contribution is the horizontally-polarized field produced by the plasmon that is coupled to the first slit and scattered by the second. We identify the mean photon number of this plasmon-induced field by $\bar{n}_{\mathrm{Pl}}$.

To obtain the combined photon distribution, we make use of the Glauber-Sudarshan theory of coherence [1, 2]. Thus, we start by writing the P-function associated to the field produced by the two independently-generated, indistinguishable horizontally-polarized modes. These represent either the photons or plasmons emerging through each of the slits, so without loss of generality, we label the P function of this combined state as

$$P_{\mathrm{Pl}}(\alpha) = \int P_1(\alpha-\alpha')\, P_2(\alpha')\, d^2\alpha', \tag{1}$$

where the P function of the thermal light fields is given by

$$P_i(\alpha) = \frac{1}{\pi\bar{n}_i}\exp\left(-\frac{|\alpha|^2}{\bar{n}_i}\right), \quad i=1,2. \tag{2}$$

The mean photon number of the two modes is represented by $\bar{n}_1 = \eta\bar{n}_{\mathrm{s}}$ and $\bar{n}_2 = \bar{n}_{\mathrm{Pl}}$, whereas α stands for the complex amplitude as defined for coherent states $|\alpha\rangle$. By substituting Supplementary Eq. (2) into Supplementary Eq. (1), we find that the P function of the combined horizontally-polarized field is given by

$$P_{\mathrm{Pl}}(\alpha) = \frac{1}{\pi(\bar{n}_1+\bar{n}_2)}\exp\left(-\frac{|\alpha|^2}{\bar{n}_1+\bar{n}_2}\right), \tag{3}$$

whose photon distribution can readily be evaluated as

$$p_{\mathrm{Pl}}(n) = \langle n|\,\rho_{\mathrm{Pl}}\,|n\rangle, \tag{4}$$

with

$$\rho_{\mathrm{Pl}} = \int P_{\mathrm{Pl}}(\alpha)\,|\alpha\rangle\langle\alpha|\, d^2\alpha. \tag{5}$$

By substituting Supplementary Eq. (5) into Supplementary Eq. (4), we can readily find that the photon distribution for the scattered photons and plasmons with horizontal polarization is given by

$$p_{\mathrm{Pl}}\left(n\right)=\frac{\left(\bar{n}_{\mathrm{Pl}}+\eta\bar{n}_{\mathrm{s}}\right)^{n}}{\left(\bar{n}_{\mathrm{Pl}}+\eta\bar{n}_{\mathrm{s}}+1\right)^{n+1}}. \tag{6}$$

We can then introduce the vertically polarized multiphoton contribution by writing the photon-number distribution at the detector as $p_{\mathrm{det}}=\sum_{m=0}^{n}p_{\mathrm{Pl}}\left(n-m\right)p_{\mathrm{Ph}}\left(m\right)$, where the distribution $p_{\mathrm{Ph}}\left(m\right)$ accounts for the vertical polarization component of the illuminating thermal field. Note that the distinguishability, i.e. statistical independence of the two thermal sources allows us to write the combined photon distribution as the convolution of the two independent probability distributions [3]. Thus, we can describe the final photon-number distribution after the plasmonic structure as

$$p_{\mathrm{det}}=\sum_{m=0}^{n}\frac{\left(\bar{n}_{\mathrm{Pl}}+\eta\bar{n}_{\mathrm{s}}\right)^{n-m}\left[\left(1-\eta\right)\bar{n}_{\mathrm{s}}\right]^{m}}{\left(\bar{n}_{\mathrm{Pl}}+\eta\bar{n}_{\mathrm{s}}+1\right)^{n-m+1}\left[\left(1-\eta\right)\bar{n}_{\mathrm{s}}+1\right]^{m+1}}, \tag{7}$$

which is the result shown in Eq. (2) of the main text. Note that Supplementary Eq. (7) is valid only when the two sources, i.e. the two slits, are active and contribute to the resulting combined field at the detector.

SUPPLEMENTARY NOTE 2. INTENSITY INDEPENDENCE OF THE MODIFICATION OF QUANTUM STATISTICS

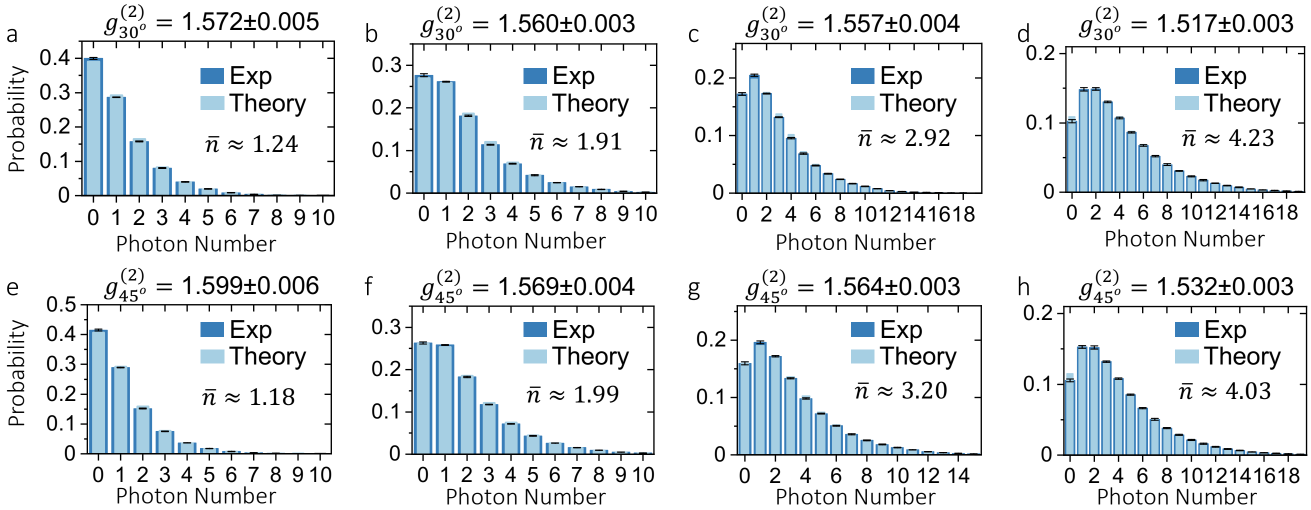

Supplementary Fig. 1. Intensity independence of the modification of quantum statistics for our plasmonic system. Panels a to d show the probability distribution and the value of the $g^{(2)}$ function for a situation in which the sample is illuminated with linearly polarized light at 30°. While the photon number distribution changes with the brightness of the source, the value of the $g^{(2)}$ remains unchained. This behavior shows the relevance of the $g^{(2)}$ function as a metric to quantify the quantum statistical fluctuations of a physical system. For sake of completeness, panels e to h show a similar trend for the case in which the sample is illuminated with diagonally polarized light. These probability distributions demonstrate that the second-order quantum coherence function $g^{(2)}$ does not change with respect to the brightness of the experiment. The error bars represent the standard deviation of ten realizations of the experiment. Each experiment consists of approximately 100000 photon-number-resolving measurements.

To further demonstrate that the second-order quantum coherence function $g^{(2)}$ does not depend on the brightness of the source, we provide additional data for the experiment reported in the Figs. 2 f and g. Here, we vary the brightness of $\bar{n}_s$ and $\bar{n}_{\mathrm{Pl}}$, while keeping the ratio $\bar{n}_s=3\bar{n}_s$ unchanged. As shown in Supplementary Figure 1, the $g^{(2)}$ indeed is independent of the mean photon number of the illuminating multiphoton system. Furthermore, we note that the theoretical calculation predicts $g^{(2)}_{30^\circ}=1.508$ and $g^{(2)}_{45^\circ}=1.531$, which is also independent of the mean photon number. As described in the main paper, this number is established by the ratio between $\bar{n}_s$ and $\bar{n}_{\mathrm{Pl}}$.

SUPPLEMENTARY NOTE 3. CHARACTERIZATION OF THERMAL LIGHT SOURCES

In our experiment, we generate pseudo-thermal light by focusing laser onto a rotating ground glass. In order to perform photon counting with our SNSPDs, we use the surjective photon counting method described in Ref. [4, 5]. The data was divided in time bins of 1 μs, which corresponds to the coherence time of our CW laser. Moreover, the 20 ns recovery time of our SNSPDs ensured that we perform measurements on a single-temporal-mode field. As shown in Supplementary Figure 2, our pseudo-thermal light mimics the photon statistics of a thermal light source.

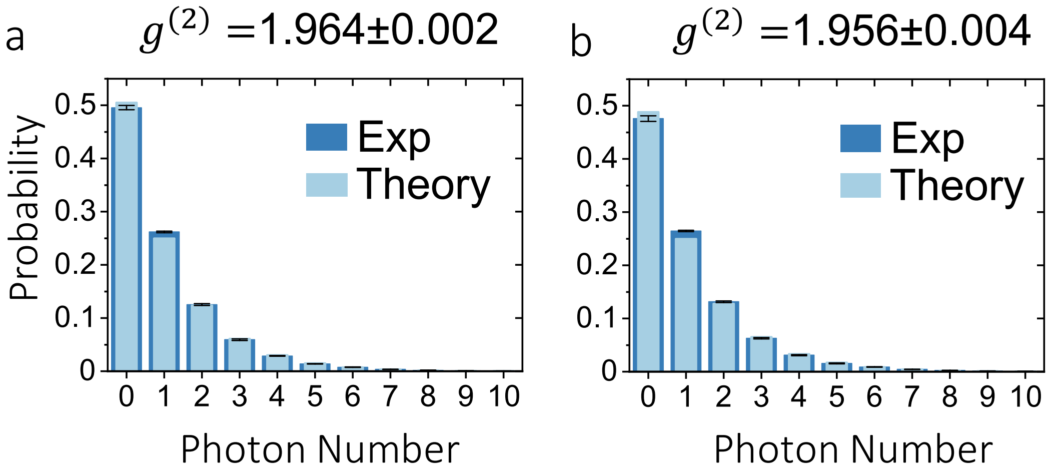

Supplementary Fig. 2. Histograms displaying theoretical and experimental photon number probability distributions for our pseudo-thermal light sources. The calculated second-order correlation functions $g^{(2)}$ certify the thermal nature of our sources. The error bars represent the standard deviation of ten realizations of the experiment. Each experiment consists of approximately 100000 photon-number-resolving measurements.

SUPPLEMENTARY NOTE 4. PHOTON STATISTICS PRODUCED BY ONE SLIT

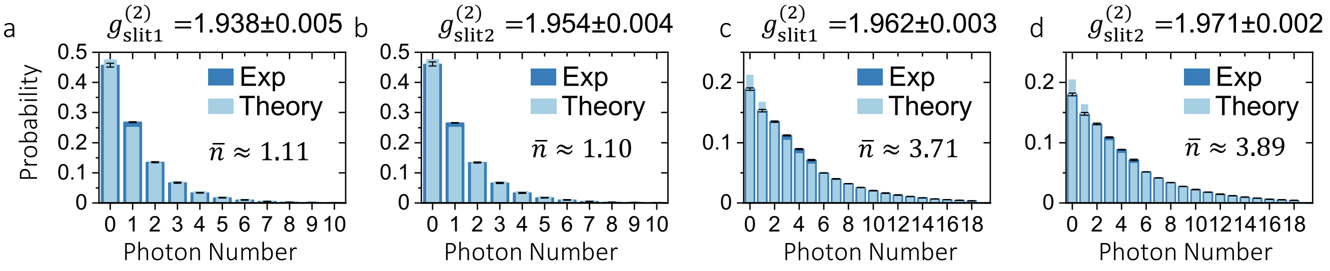

Supplementary Fig. 3. Histograms displaying theoretical and experimental photon-number probability distributions of the output from either slit. In a and c, the second slit, where the SPPs are transmitted, is blocked. Therefore, the probability distribution corresponds to the transmitted thermal beam. In b and d, the first slit is blocked, thus the probability distribution represents the quantum statistics of the plasmonic mode. The error bars represent the standard deviation of ten realizations of the experiment. Each experiment consists of approximately 100000 photon-number-resolving measurements.

Here, we discuss the photon statistics produced by a single plasmonic slit. For this purpose, we measure the photon statistics when blocking either slits in our plasmonic structure. In Supplementary Figure 3, we plot the measured photon number distributions when blocking either slit. As expected, when measuring the transmitted light from the first slit (while keeping the second blocked), $g^{(2)}_{\text{slit1}}$ is close to 2, which is similar to the $g^{(2)}$ of our thermal source. Similarly, when measuring only the second slit (while keeping the first blocked), where the SPPs are generated, the $g^{(2)}_{\text{slit2}}$

is also close to 2. As discussed in the main text of the manuscript, these results validate that the plasmonic structure indeed preserves quantum statistics through the simple single-particle dynamics. However, the additional scattering paths supported by our sample induce complex multiparticle interactions, the resulting multiparticle dynamics can, in turn, lead to the modification of the excitation mode of plasmonic systems. Furthermore, we plot the measured photon-number distributions for the first slit with different polarizations. As shown in Supplementary Figure 4, the $g^{(2)}_{\text{slit1}}$ is still close to 2, and is independent of the polarization angle. Note that the theoretical prediction in Supplementary Figure 4 is obtained by using the analytical expression for a thermal photon distribution, rather than Supplementary Eq. (7). This shows that one cannot describe the statistics of the photons emitted by a single slit as the statistical combination of two independent polarized fields. More importantly, this result supports our central claim, which states that it is only when a plasmon—i.e., a second field source—is excited that photon statistics of the initially thermal field are modified.

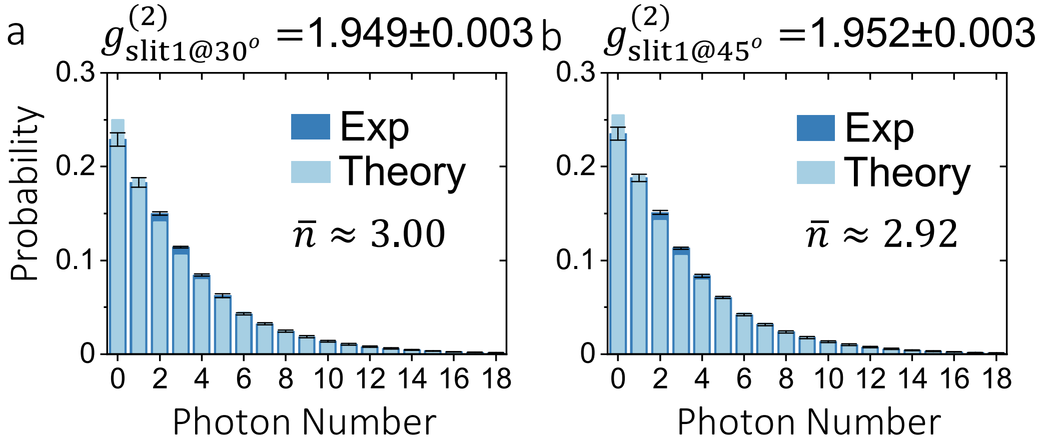

Supplementary Fig. 4. Histograms displaying theoretical and experimental photon-number probability distributions of the output from the first slit. The probability distribution corresponds to the transmitted thermal beam, and it is independent from the beam's polarization. The error bars represent the standard deviation of ten realizations of the experiment. Each experiment consists of approximately 100000 photon-number-resolving measurements.

[1] R. J. Glauber, Coherent and incoherent states of the radiation field, Phys. Rev. **131**, 2766 (1963).

[2] E. C. G. Sudarshan, Equivalence of semiclassical and quantum mechanical descriptions of statistical light beams, Phys. Rev. Lett. **10**, 277 (1963).

[3] R. V. Hogg, J. McKean, and A. T. Craig, *Introduction to mathematical statistics* (Pearson Education, 2005).

[4] S. M. H. Rafsanjani, M. Mirhosseini, O. S. Magaña-Loaiza, B. T. Gard, R. Birrittella, B. E. Koltenbah, C. G. Parazzoli, B. A. Capron, C. C. Gerry, J. P. Dowling, and R. W. Boyd, Quantum-enhanced interferometry with weak thermal light, Optica **4**, 487 (2017).

[5] C. You, M. A. Quiroz-Juárez, A. Lambert, N. Bhusal, C. Dong, A. Perez-Leija, A. Javaid, R. d. J. León-Montiel, and O. S. Magaña-Loaiza, Identification of light sources using machine learning, Appl. Phys. Rev. **7**, 021404 (2020).
